# Development of triptolide-nanoemulsion gels for percutaneous administration: physicochemical, transport, pharmacokinetic and pharmacodynamic characteristics

**DOI:** 10.1186/s12951-017-0323-0

**Published:** 2017-12-04

**Authors:** Meng Yang, Yongwei Gu, Dishun Yang, Xiaomeng Tang, Jiyong Liu

**Affiliations:** 1 Department of Pharmacy, Changhai Hospital, Second Military Medical University, Shanghai, 200433 China; 2 Department of Pharmacy, Shandong University of Traditional Chinese Medicine, Jinan, 250355 China

**Keywords:** Triptolide, Nanoemulsions, Nanoemulsion gels, Transdermal drug delivery system, Skin-vessel synchronous microdialysis, Chronic dermatitis/eczema model

## Abstract

**Background:**

This work aimed to provide useful information on the use of nanoemulsions for the percutaneous administration of triptolide. Lipid nanosystems have great potential for transdermal drug delivery. Nanoemulsions and nanoemulsion gels were prepared to enhance percutaneous permeation. Microstructure and in vitro/in vivo percutaneous delivery characteristics of triptolide (TPL)-nanoemulsions and TPL-nanoemulsion gels were compared. The integrity of the nanoemulsions and nanoemulsion gels during transdermal delivery and its effects on the surface of skin were also investigated. The penetration mechanisms of nanoemulsions and nanoemulsion gels were investigated by differential scanning calorimetry (DSC) and Fourier transform infrared spectroscopy (FTIR). The transport characteristics of fluorescence-labelled nanoemulsions were probed using laser scanning confocal microscopy. A chronic dermatitis/eczema model in mice ears and the pharmacodynamic of the TPL-nanoemulsion gels were also investigated.

**Results:**

Compared to TPL gels, significantly greater cumulative amounts of TPL-nanoemulsion gels and TPL-nanoemulsions penetrated rat skin in vitro. The in vivo microdialysis showed the concentration–time curve AUC_0–t_ for TPL-NPs is bigger than the TPL-gels. At the same time, TPL-NPs had a larger effect on the surface of skin. By hydrating keratin and changing the structure of both the stratum corneum lipids and keratin, nanoemulsions and nanoemulsion gels influence skin to promote percutaneous drug penetration. Both hairfollicles and the stratum corneum are also important in this transdermal drug delivery system. Moderate and high dosages of the TPL-nanoemulsion gels can significantly improve the symptoms of dermatitis/eczema inflammation and edema erythematic in mice ears and can reduce the expression of IFN-γ and IL-4. Moreover, the TPL-nanoemulsion gels cause less gastrointestinal damage than that of the *Tripterygium wilfordii* oral tablet does.

**Conclusions:**

Nanoemulsions could be suitable for transdermal stably releasing drugs and maintaining the effective drug concentration. The TPL-nanoemulsion gels provided higher percutaneous amounts than other carriers did. These findings suggest that nanoemulsion gels could be promising percutaneous carriers for TPL. The TPL-nanoemulsion gels have a significant treatment effect on dermatitis/eczema in the mice model and is expected to provide a new, low-toxicity and long-term preparation for the clinical treatment of dermatitis/eczema in transdermal drug delivery systems.

**Electronic supplementary material:**

The online version of this article (10.1186/s12951-017-0323-0) contains supplementary material, which is available to authorized users.

## Background

Triptolide belongs to the Celastraceae family and is used extensively as an antirheumatoid, anti-eczema, immunosuppressive, nephrotic syndrome and malignant tumour agent in traditional Chinese medicine. The pharmacological effects of TPL are attributed to its anti-inflammatory, analgesic, anti-tumor and immune-regulating properties [1]. However, the oral intake of TPL exists in a high-toxicity region, and the digestive system and urogenital system have adverse reaction rates of 35.8 and 22.8%, respectively [2], severely limiting its clinical application. Transdermal drug delivery is an important approach to effectively reduce these adverse reactions. Transdermal delivery is superior to oral administration as a route for TPL because it avoids the first-pass metabolism in liver, reducing its oral drug damage to the gastrointestinal tract, liver and kidney [3]. However, its composition is complicated by poor water solubility and difficult entry through the skin, which affects the extent of its curative effect and the pharmacokinetics [4] of its metabolic conversion in the skin, for which both of the mechanisms are unclear. O/W nanoemulsion gels, used as transdermal drug delivery carriers, can enhance a drug’s solubility and can move it easily through the skin, improving its efficiency. Additionally, nanoemulsion gels can control the drug’s release, maintaining stable and lasting blood drug concentrations [5].

Lipid-based nanosystems are relatively new nanocarriers that are used for transdermal drug delivery. Nanoemulsions is a kind of transparent or semi-transparent and thermodynamic unstable system formed by an appropriate proportion mixture of mixing oil phase, aqueous phase, surfactant and co-surfactant [6]. Nanoemulsions can’t form spontaneously and require external shear to overcome surface tension. Compared to the others formulates, the nanoemulsions could maintain a longer stability in the process of storage and application. Higher stability and better solubility of solutes are also the characteristics of nanoemulsions, and the drugs may be loaded in the internal phase or distribute in the external phase of nanoemulsions [7]. The W/O nanoemulsions can extend the water-soluble drugs release time and effect, O/W nanoemulsions can increase the lipophilicity drug solubility and the B.C nanoemulsions is the transition states between the W/O and O/W, which is little practical application [8]. Nanoemulsions and nanoemulsion gels are two types of nanocarriers that have been shown to enhance transdermal permeation. Compared with other transdermal vehicles such as gels, creams, tinctures, and lipid-gels, they enhance drug solubility and have a simple preparation process, a good physical stability, a low viscosity, a good targeting effect and a controlled release for long-term effects. However, the facts that the lack of adhesion with skin and water phase evaporating in the storage process and that the skin irritation all need further studies for the application of nanoemulsions. Alves etc. put forward the concept of nanoemulsion gels which was prepared by nanoemulsions with a proportional gel matrix [9]. Nanoemulsion gels have the beneficial properties of nanoemulsions but overcome their limitations, including chemical instability and skin irritation. Furthermore, nanoemulsion gels have been confirmed to produce efficient transdermal drug permeation.

A number of strategies were carried out to investigate the pharmacokinetic of TPL formulations and to obtain more reasonable treatment of this active compound. In this study, we prepared and evaluated TPL-nanoemulsions and TPL-nanoemulsion gels, to observe them physicochemical, transport, pharmacokinetic and pharmacodynamic characteristics in blood and in skin as percutaneous administration.

## Results

### Preparation and characterization of nanoemulsions and nanoemulsion gels [10]

The optimal procedure for the preparation of the TPL-nanoemulsions included 0.025% triptolide, 18% Capryol 90, 30% OP-10, 15% 1,2-propylene glycol and 37% water. The particle size and PDI are important characteristics of nanoemulsions and nanoemulsion gels that describe the distribution of nanoparticles [11]. The nanoemulsions and nanoemulsion gels had PDI values of 0.13 ± 0.018 and 0.19 ± 0.023, respectively, and narrow size distributions of 45.6 ± 1.7 and 62.1 ± 9.9 nm, respectively. Additionally, the DL of the nanoemulsions and nanoemulsion gels associated with TPL were 0.025 ± 0.30 and 0.0245 ± 0.33%, respectively.

**Fig. 1:**
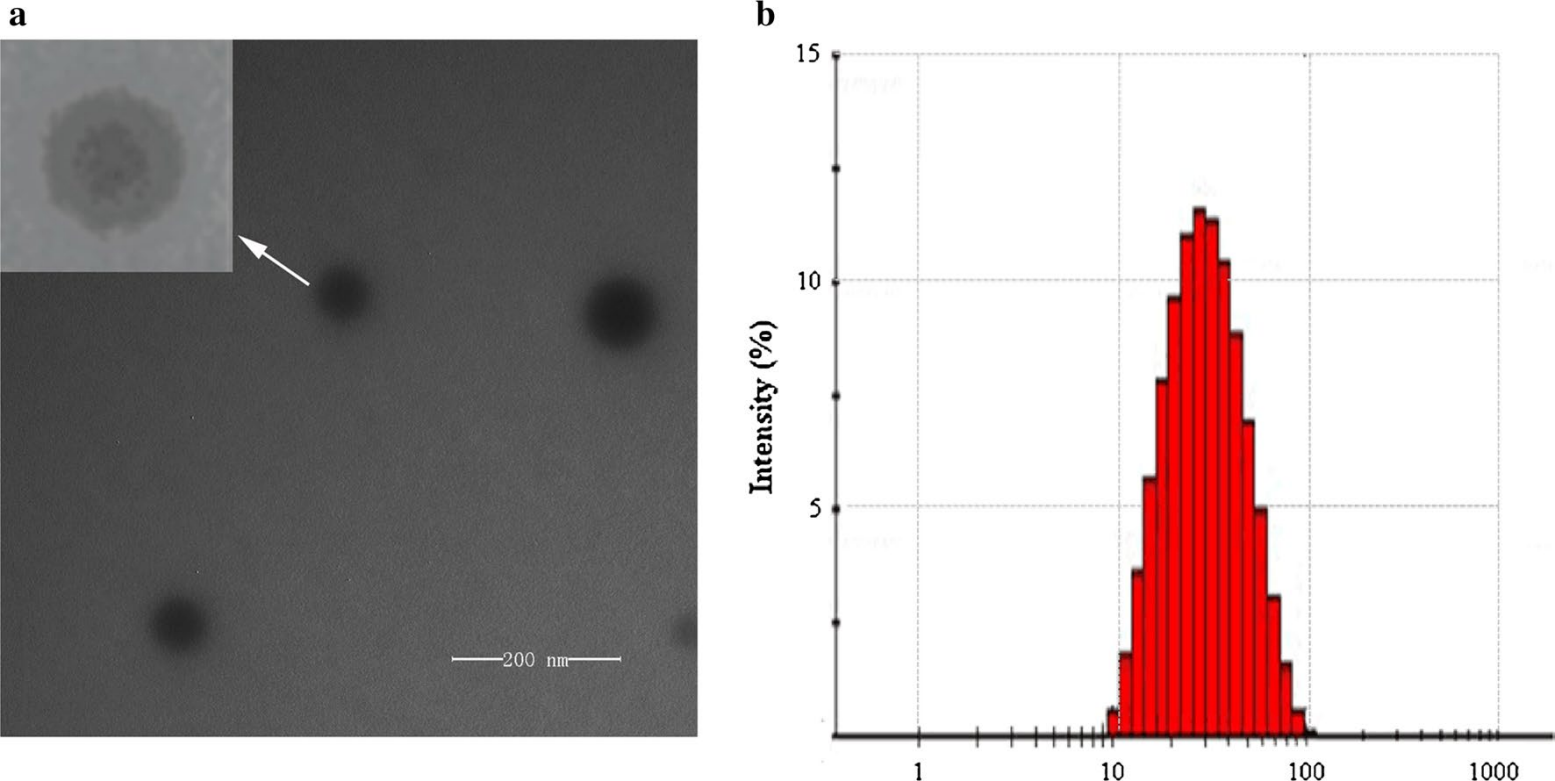
TEM image and size distribution of the TPL nanoemulsions (**a** TEM, ×50,000; **b** size distribution)

The morphologies of the TPL-nanoemulsions and TPL-nanoemulsion gels were observed using transmission electron microscopy (TEM). As shown in Fig. 1, the nanoemulsions have uniform spheres and an obvious two-layered structure. Together, the core and the surrounding external parts is approximately 30 nm. A roughly normal distribution is observed for the particle size, showing that it meets the requirements of nanoemulsions and that the preparation has good uniformity [12].

### In vitro release

**Fig. 2:**
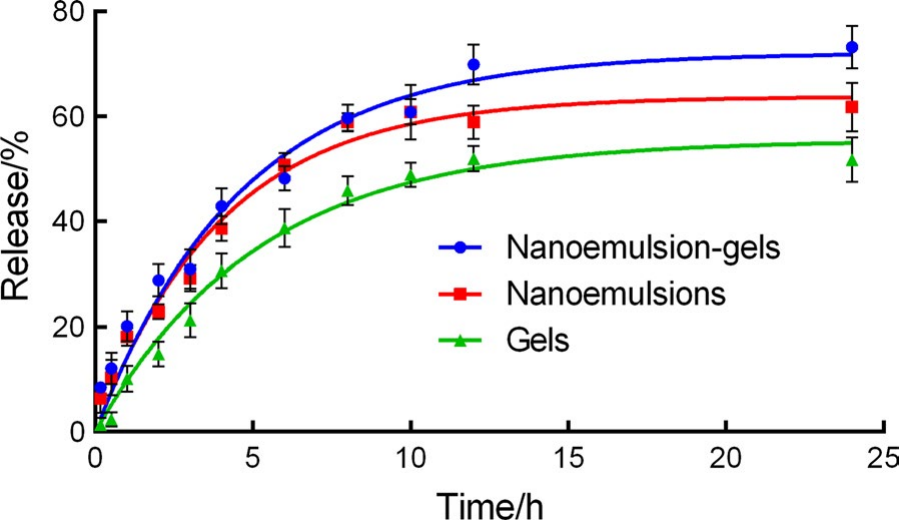
In vitro release profiles of TPL-nanoemulsions, nanoemulsion-gels and gels (n = 3)

The cumulative release percentage profiles of TPL from the TPL-nanoemulsions, the TPL-nanoemulsion gels and the TPL-gels are shown in Fig. 2. Both the nanoemulsion gels and the nanoemulsions dispersions significantly enhanced the in vitro TPL release for 24 h. The nanoemulsion gels, the nanoemulsions and the gels formulations show a controlled release with a sudden release at 12, 6 or 8 h, respectively (of 70, 56 and 48%, respectively). The results showed the TPL dispersions from nanoemulsion gels, the nanoemulsions and the ordinary gels were 76.2 ± 2.0, 61.2 ± 0.4 and 53.2 ± 0.4, respectively. The total amounts of the drug that releases from the nano-formulations were slightly greater than that released from the ordinary gels.

### In vitro percutaneous permeation

**Fig. 3:**
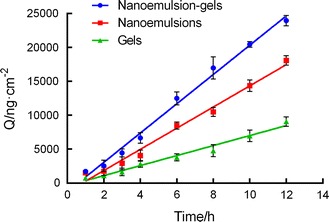
Transdermal characterization of the TPL-nanoemulsions, the nanoemulsion gels and the gels in vitro (n = 5)

In this study, the nanoemulsion gels enhanced the transdermal permeation of TPL (as shown in Fig. 3) more effectively than the nanoemulsions did. Initially, TPL was released rapidly from both the nanoemulsions and the nanoemulsion gels, but they later showed a sustained release, which is consistent with the in vitro release study. The results shown in Table 1 include the cumulative osmotic quantity and penetration rate of the 4 drug delivery systems, which are ranked as follows: TPL-nanoemulsion gels > TPL-nanoemulsions > TPL-gels > TPL-saturated aqueous solution. The triptolide saturated aqueous solution has difficulty passing through the skin, and preparation of the drug into the nano-formulations enhances the percutaneous absorption of the drug [13].

**Table 1:** Comparison of the different doses in the transdermal test (n = 5)

System	Regression equation	Qn/ng/cm^2^	Js/ng/cm^2^/h
TPL-saturated aqueous solution	ND	ND	ND
TPL-gels	y = 737.8x − 377.5	9070.45	737.8
TPL-nanoemulsions	y = 1560x − 1272	18,071.94	1560
TPL-nanoemulsion-gels	y = 2163x − 1316	23,945.68	782.16

### In vivo skin-vessel synchronous microdialysis

**Fig. 4:**
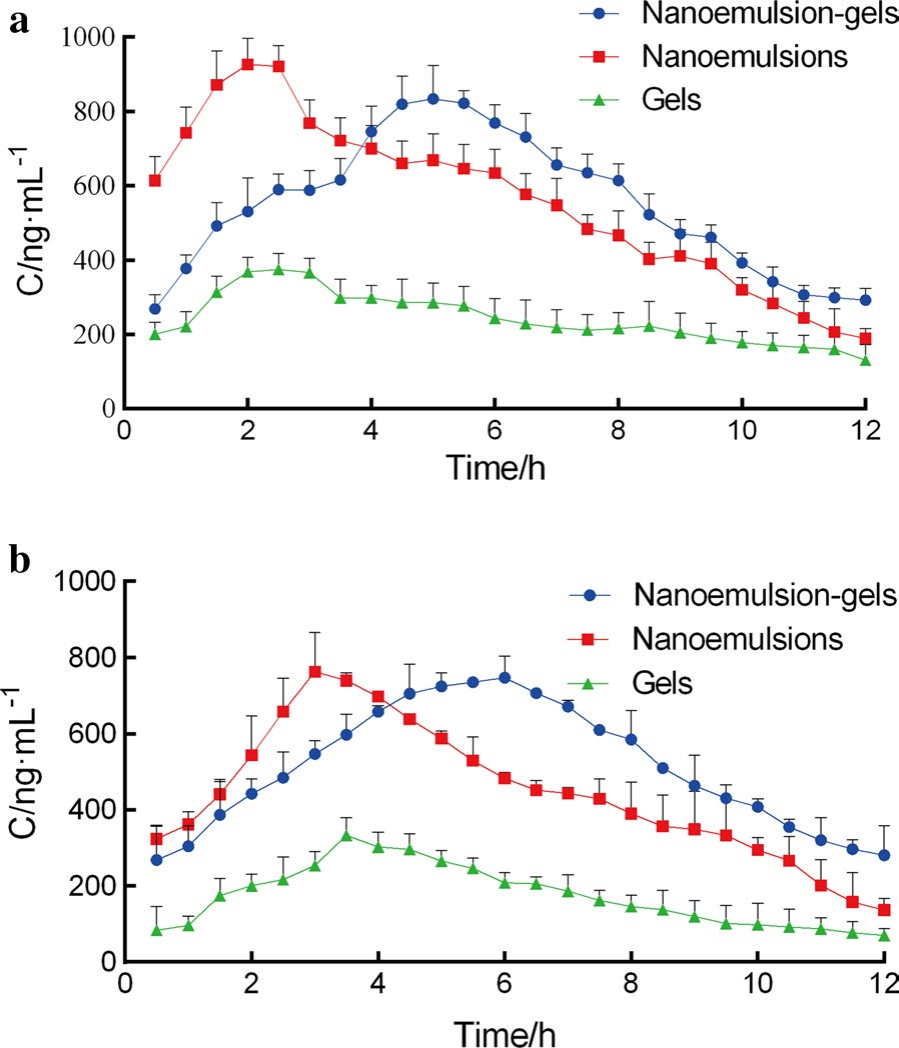
Concentration–time profiles of triptolide in microdialysates of skin (**a**) and blood (**b**) after the application of the TPL transdermal delivery systems (n = 3)

In vivo time-concentration profiles of TPL after topical application of the TPL-nanoemulsions and the TPL-nanoemulsion gels on the skin of rats are depicted in Fig. 4, and the resultant pharmacokinetic parameters are summarized in Table 2. The peak concentrations (C_max_) of the TPL-nanoemulsions and the TPL-nanoemulsion gels were similar at approximately 6 h after administration. The C_max_ were 833.75, 976.21 and 375.15 ng/mL (for skin) and 747.41, 762.98 and 312.75 ng/mL (for blood) for the samples treated with the TPL-nanoemulsion gels, TPL-nanoemulsions and the TPL-gels, respectively. The areas under the concentration–time curves (AUC_0–t_) of the nanoemulsion gels and the nanoemulsions were 2.26- and 2.25-fold higher in the skin, respectively (2.93- and 2.54-fold higher in the blood, respectively) than that of the TPL-gels. The TPL-gels had difficulty passing through the skin, causing the skin and blood concentrations to be lower than that of the TPL-nanoemulsions and the TPL-nanoemulsion gels, which have better transdermal effects. Compared with the TPL-nanoemulsions, the TPL-nanoemulsion gels has a higher AUC in the skin and blood after the percutaneous drug delivery, and the drug concentration is released more smoothly, providing a sustained release effect and an improved bioavailability [14].

**Table 2:** Pharmacokinetic parameters in skin and blood after the application of the TPL transdermal delivery systems (n = 3)

Parameters	Skin			Blood		
	Gels	Nanoemulsions	Nano-gels	Gels	Nanoemulsions	Nano-gels
C_max_ (ng/mL)	375.15 ± 5.53	976.21 ± 3.34	833.75 ± 3.75	312.75 ± 4.49	762.98 ± 8.64	747.41 ± 7.72
T_max_ (h)	2.50 ± 0.86	2.00 ± 0.43	5.00 ± 0.99	3.50 ± 1.21	3.00 ± 1.04	6.00 ± 2.03
T_1/2_ (h)	7.45 ± 1.21	2.55 ± 0.12	14.58 ± 2.35	3.67 ± 0.47	0.70 ± 0.11	3.85 ± 0.51
MRT	11.55 ± 1.43	6.06 ± 1.05	19.04 ± 1.96	7.18 ± 1.58	5.82 ± 0.77	8.31 ± 2.12
AUC_0–720_ (ng h/mL)	2882.41 ± 11.61	6523.17 ± 30.13	6512.53 ± 30.97	2062.15 ± 14.78	5248.94 ± 43.15	6045.41 ± 50.97
AUC_0–∞_ (ng h/mL)	4454.43 ± 20.21	7208.27 ± 40.65	12,660.87 ± 50.12	2428.88 ± 16.95	5576.44 ± 47.96	7569.36 ± 64.31

### Penetration mechanisms analysis

**Fig. 5:**
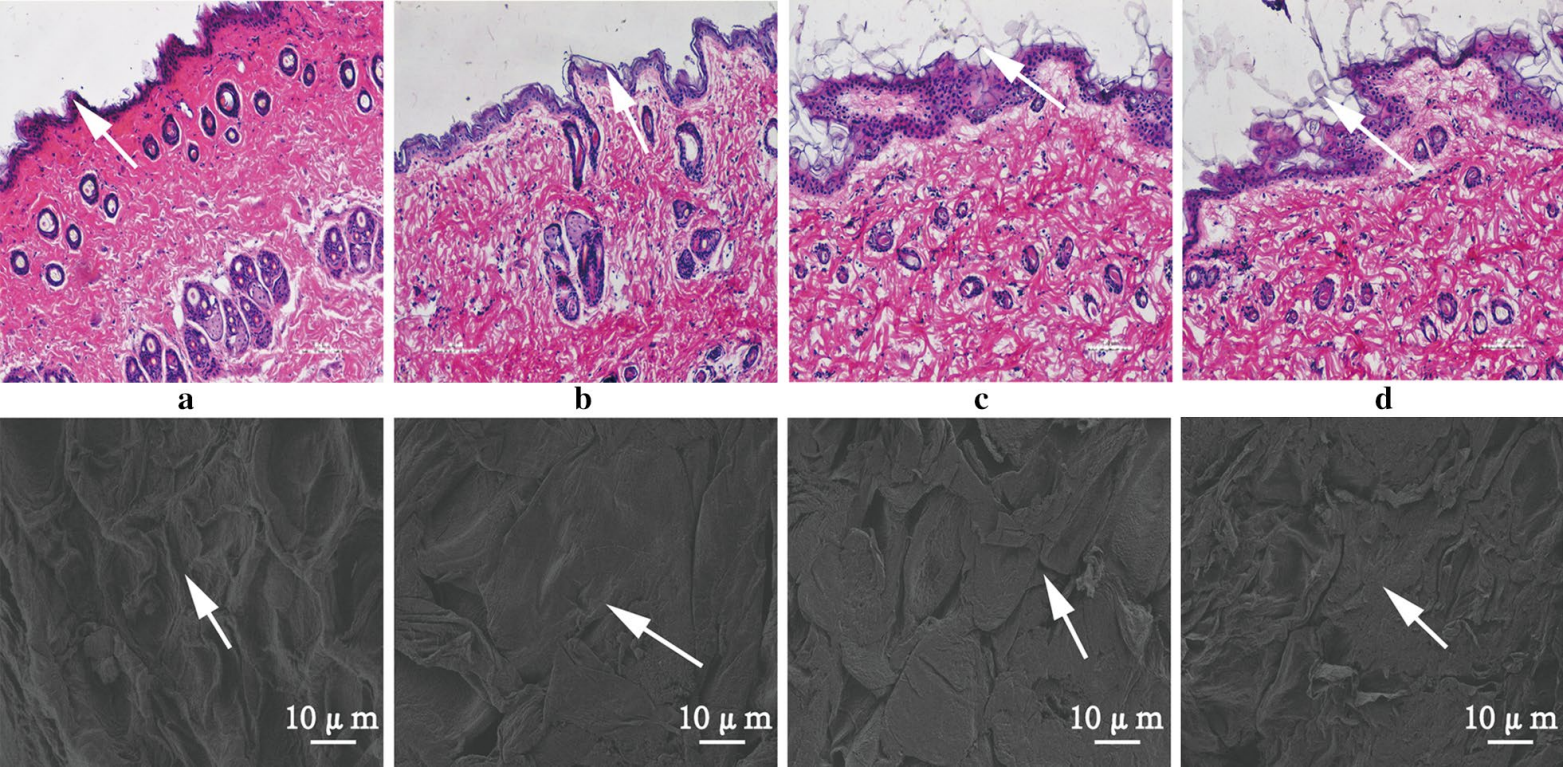
Histopathological microscopic slides and microstructure of the rat skin. Skin samples with normal control (**a**), TPL-gels-treated (**b**), TPL-nanoemulsions-treated (**c**), TPL-nanoemulsion gels-treated (**d**) obtained via HE dyeing method and SEM (×1000 magnification)

Histopathological analysis of microscopic slides of the rat skin (Fig. 5) shows that the surface of the control rat skin (without any treatment) had a tightly compacted stratum corneum that was smooth and intact (Fig. 5a). However, the surface of the skin exposed to the TPL-gels (Fig. 5b), the TPL-nanoemulsions (Fig. 5c) or the TPL-nanoemulsion gels (Fig. 5d) formulation showed a fall-off phenomenon, which includes thinning of the stratum corneum and widening of its cracks.

The treated rat skin was observed by scanning electron microscopy (SEM) to inspect the effect of the different formulations on the structure of the stratum corneum. Compared with the control group, the TPL-gels made the corneous layer loose and irregular, while the TPL-nanoemulsions resulted in a curly and deformed stratum corneum and the TPL-nanoemulsion gels causes the cuticle to further fall off the skin.

**Fig. 6:**
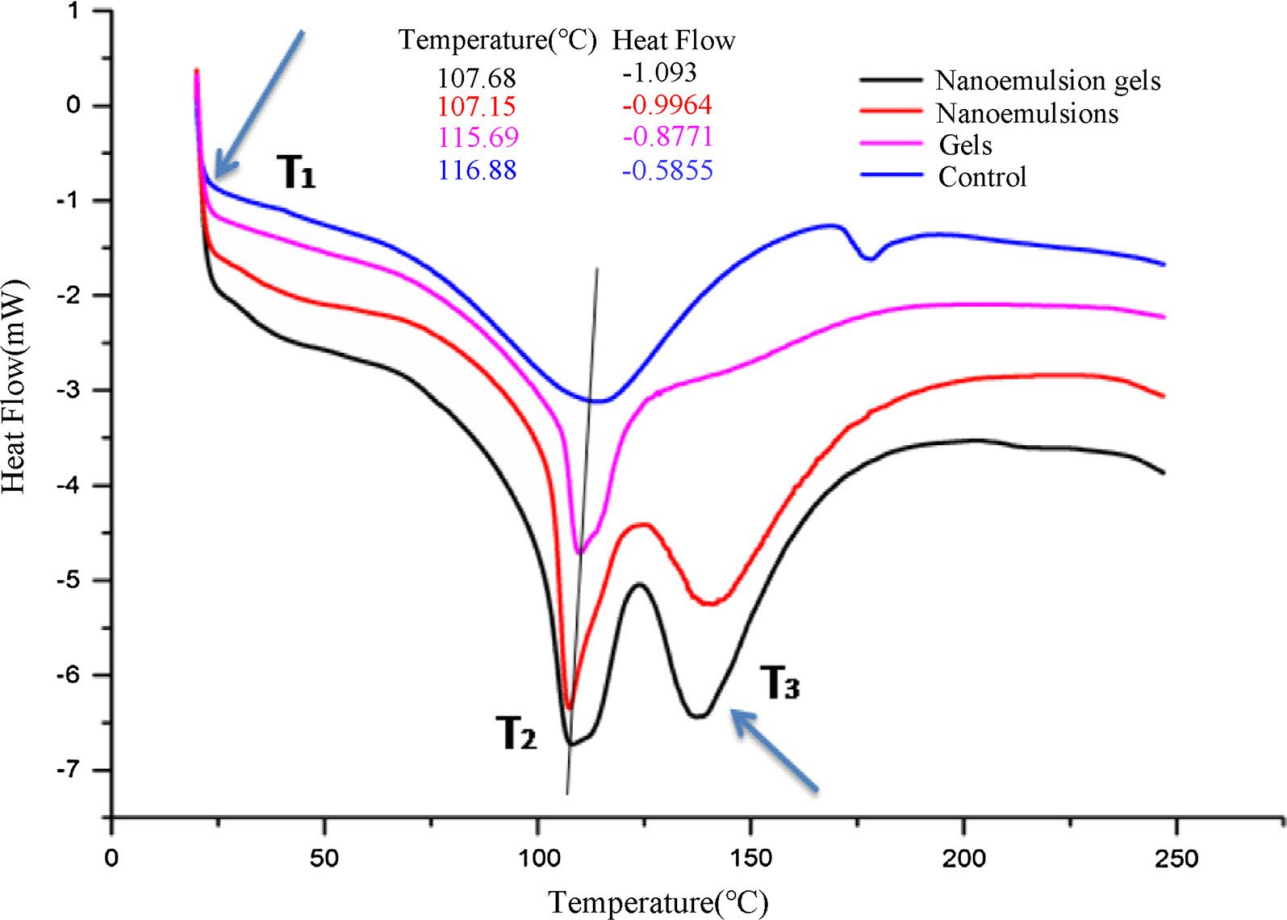
DSC Thermograms of skin treated with a normal control, the TPL-gels, the TPL-nanoemulsions and the TPL-nanoemulsion gels

The influences of each formulation on the stratum corneum were investigated by DSC [15] and FTIR [16]. Thermograms corresponding to reference data from rat skin treated with the normal control, the TPL-gels, the TPL-nanoemulsions and the TPL-nanoemulsion gels are shown in Fig. 6. This study shows three endothermic peaks: T_1_, T_2_ and T_3_. After the formulations were applied, T_1_ showed a gradual decline, indicating that the formulation changed the structure of the lipids in the skin and improved the liquidity. T_2_ (as shown) was caused by the denaturation of cell proteins, and in contrast with the control group, the gels-treated group showed degeneration of the stratum corneum proteins. The keratin in the nanoemulsions-treated group degraded substantially, whereas the keratin of the nanoemulsions-treated group degraded the most. T_3_ was the high-temperature absorption peak, and with increases in the water content, the endothermic peak became sharp and shifted toward lower temperatures. The T_3_ peak of the control group was 178.16 °C, which fell to 137.85 °C for the group treated with the TPL-nanoemulsion gels.

**Fig. 7:**
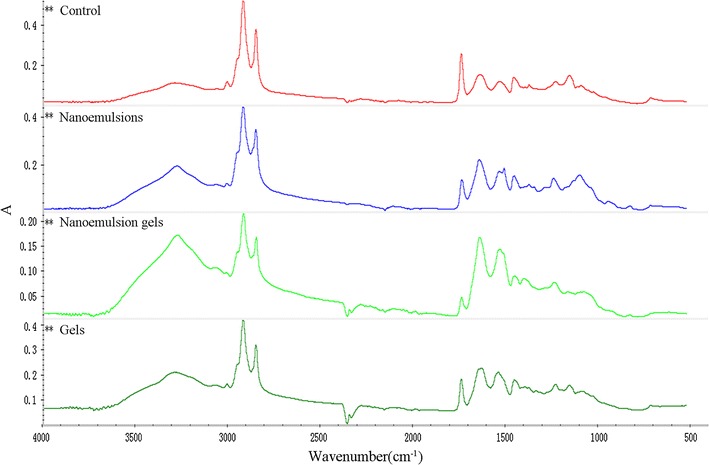
FTIR spectra of the stratum corneum treated with the normal control, TPL-gels, TPL-nanoemulsions and TPL-nanoemulsion gels

The lipids in the stratum corneum appeared in the infrared spectrum at characteristic peaks between 3000 and 2800 cm^−1^, which mainly correspond with lipid CH_2_ symmetric and asymmetric stretching vibrations. The asymmetric stretching vibration frequency of CH_2_ was between 2920 and 2930 cm^−1^, and the symmetric stretching vibration frequency was between 2850 and 2860 cm^−1^ [17, 18]. As Fig. 7, after the formulations acted on the skin, the lipid structure in the stratum corneum became disordered, this weakened the CH_2_ symmetric and asymmetric stretching vibration peak. This implies awaked lipid barrier in the stratum corneum, especially for the TPL-nanoemulsion gels group but also for the TPL-nanoemulsions group, and, least of all, for the TPL-gels group. Typical hydration of the corneous layer is characterized by strong amino (1500–1700 cm^−1^) and water (3000–3600 cm^−1^) peaks. The characteristic peaks of the amide I and amide II vibrations (1600–1700 and 1500–1600 cm^−1^, respectively) were due to the keratin in the stratum corneum. The vibration of the amide I from 1647 to 1636 cm^−1^, and the vibration of the amide II from 1545 to 1513 cm^−1^ suggest that the corneous layer of keratin may transition from an alpha helix to a random coil. The symmetric stretching vibration frequency of O–H was between 3200 and 3300 cm^−1^, and the substantial increase in the O–H vibration from 3293 to 3276 cm^−1^ suggests that keratin became hydrated. Both keratin hydration and the structural changes to the lipids and keratin can reduce the barrier function of the stratum corneum and thus promote the percutaneous penetration of the drug. Three fomulations FTIR peak areas of methylene, carbonyl, amide I and amide II were shown in Table 3.

**Table 3:** Peak areas of methylene, carbonyl, amide I and amide II in the FTIR spectra

Group	Symmetric O–H	Asymmetric C–H	Symmetric C–H	Amide I	Amide II
Control	36.290	29.429	24.414	22.733	19.951
TPL-gels	58.279	29.424	22.893	21.627	18.792
TPL-nanoemulsions	61.341	20.976	17.892	17.044	11.954
TPL-nanoemulsion gels	74.962	11.092	9.746	13.968	6.226

### Transport characteristic research

**Fig. 8:**
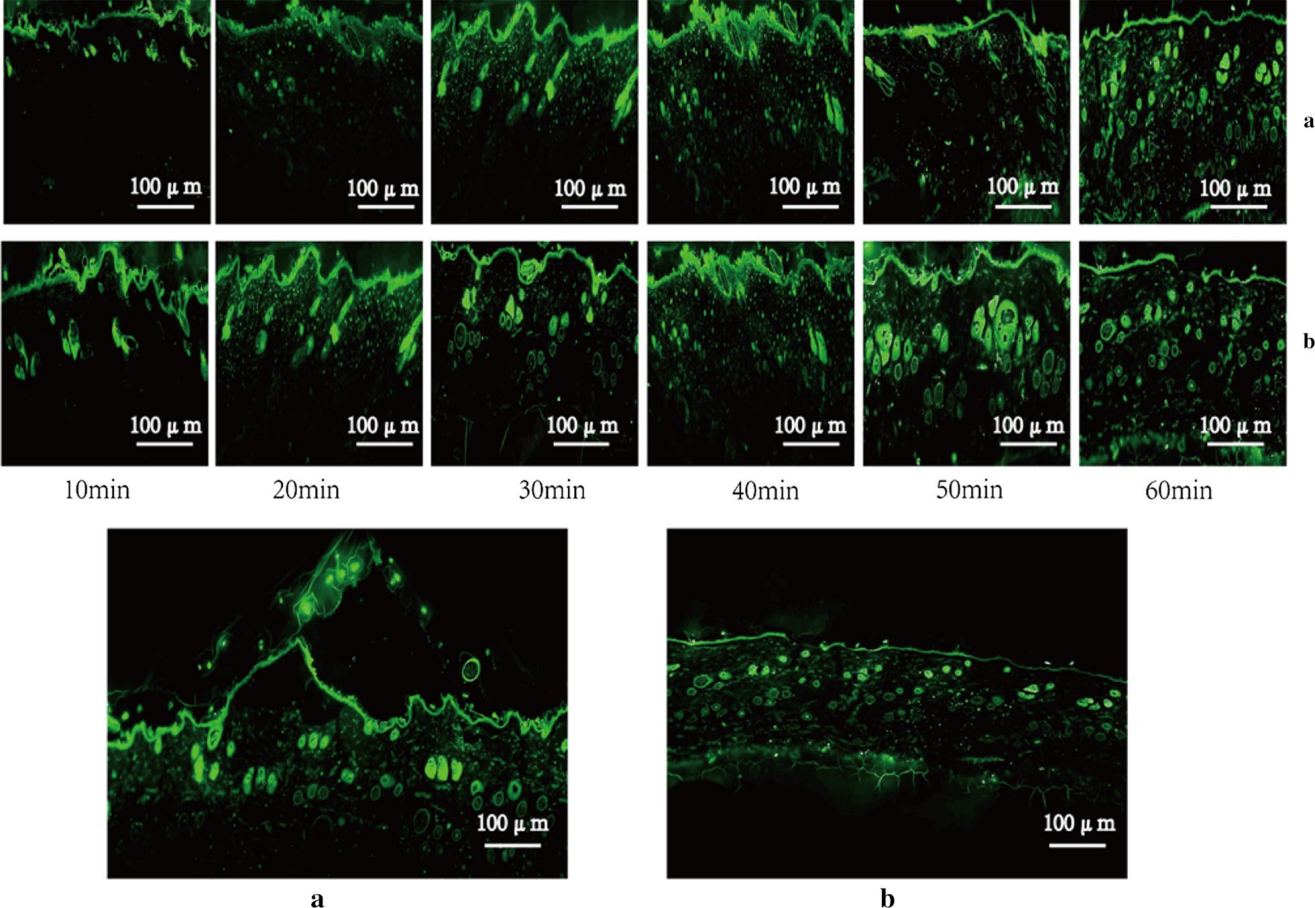
Fluorescence micrographs of stratum corneum treated with TPL-NPs with coumarin-6 fluorescent probes (**a** coumarin-6TPL-nanoemulsion gels; **b** coumarin-6TPL-nanoemulsions)

Figure 8 shows the fluorescence photomicrograph of skin treated with coumarin-6-TPL-NPs fluorescent probes. The fluorescence was mainly detected in the stratum corneum at first, with no evidence of diffusion into the epidermis. Coumarin-6 fluorescence was primarily localized in the hair follicles as well as in the stratum corneum. After 30 min, there was a more significant fluorescence in the hair follicles treated with either the coumarin-6-TPL-nanoemulsion gels or the coumarin-6-TPL-nanoemulsions. It is clear that coumarin-6 fluorescence was largely retained in the hair after laser exposure, revealing that substantial amounts of the drug passed through skin appendant organs such as hair follicles, to percutaneous absorption [19]. At longer durations, a broad and continuous fluorescence band extended from the stratum corneum into the deeper layers of the epidermis [20]. A similar result was both observed for the A and B groups, but the B group reached the epidermis before the A group, and the A group caused the stratum corneum to show a fall-off phenomenon.

### Pharmacodynamic study

**Fig. 9:**
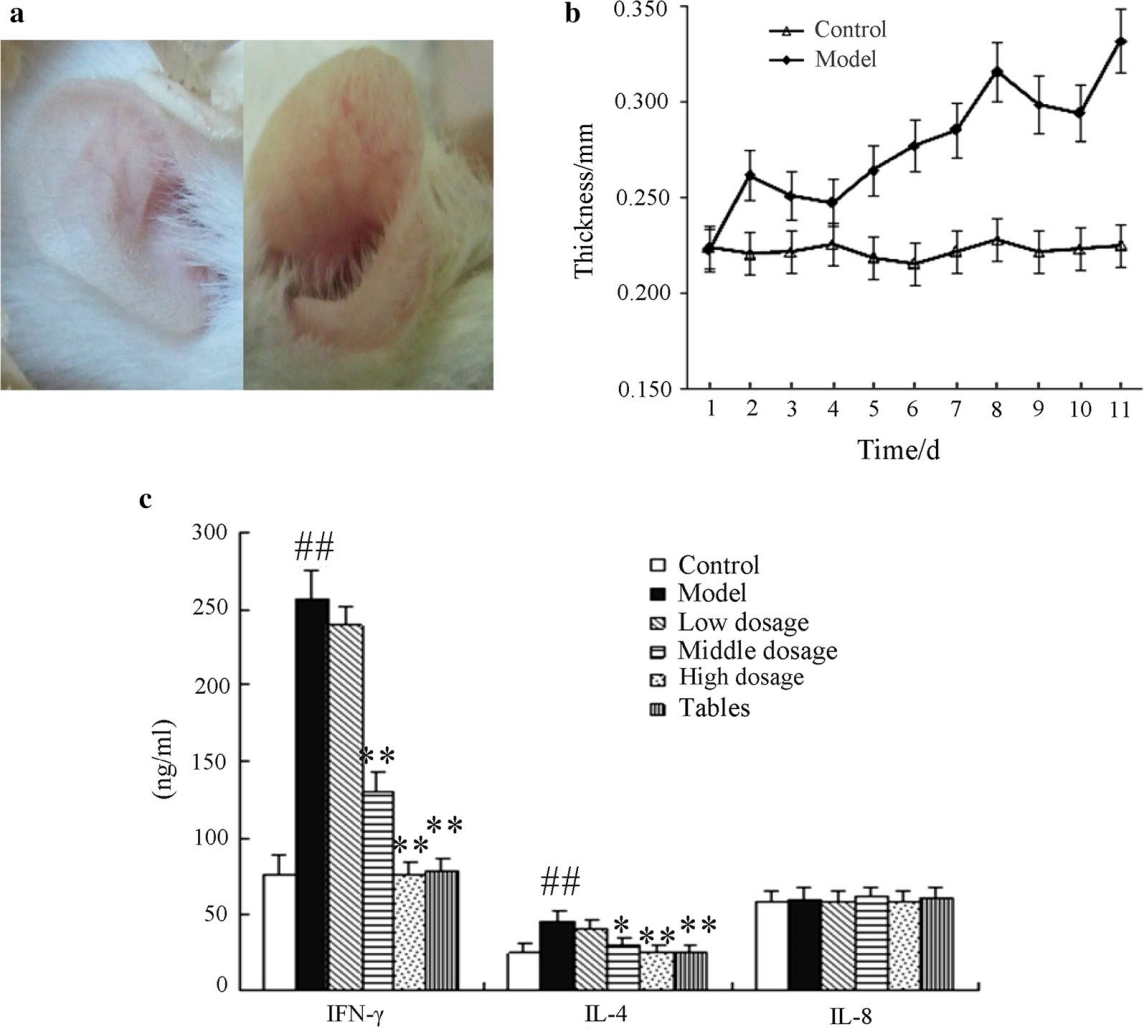
Chronic dermatitis/eczema mice model (**a** Before and after of building the chronic dermatitis/eczema model in rat’s ears: left is before and right is after. **b** Change in the ear thickness in the chronic dermatitis/eczema mice model. **c** The influence of cytokine expression in the chronic dermatitis/eczema mice model caused by the TPL-nanoemulsion gels)

According to the earlier report that established the dermatitis/eczema model in rats, ear symptoms such as swelling and thickening are common after building the model [21, 22], as shown in Fig. 9a, b. After the last excitation, the thickness of the rat’s ears in the control and model group were 0.225 ± 0.010 and 0.332 ± 0.036 mm (P < 0.01), respectively, indicating that the model was successful. ELISA was used to measure the cytokine concentration of IFN-γ, IL-4 and IL-8 in the animal serum. The results show that the concentrations of IFN-γ and IL-4 were significantly increased in model group. Moderate and high doses of the TPL-nanoemulsion gels can significantly reduce IFN-γ and IL-4 expression. The concentration of IFN-γ was reduced from 257.17 ± 8.48 to 129.79 ± 3.41 and 76.46 ± 3.19 ng/mL for the moderate and high doses, respectively (P < 0.01), and the concentration of IL-4 reduced from 45.54 ± 1.77 to 29.68 ± 1.60 and 25.01 ± 1.57 ng/mL, respectively (P < 0.01); *Tripterygium wilfordii* tablets also show an obvious inhibitory effect on IFN-γ and IL-4. However, the model, TPL-nanoemulsion gels and the *Tripterygium wilfordii* tablets have no significant influence on IL-8 expression. The results are shown in Fig. 9c.

The thickness of ear was measured by a thickness gauge, scored by macroscopic observation, and graded against a standard that was obtained from the literature. Results showed that when compared with model group, medium and high doses of the TPL-nanoemulsion gels could significantly inhibit the degree of mice ear swelling (P < 0.01). The positive control drug also significantly inhibited the degree of mice ear swelling and skin erythematic; its role had no significant difference with the TPL-nanoemulsion gels. The low-dose group showed no significant difference compared with the model group. These results are shown in Table 4. They show that the moderate- and high-dose groups of the TPL-nanoemulsion gels can significantly improve the symptoms of dermatitis/eczema inflammation and edema erythematic in mice ears.

**Table 4:** The effects of the triptolide nanoemulsion gels on the chronic dermatitis/eczema mice model ($\overline{x}$ ± s, n = 12, mm)

Score group	First			Second			Third			Fourth
	d1	d2	d3	d1	d2	d3	d1	d2	d3	d1
Model	1.23 ± 0.53	1.11 ± 0.66	1.02 ± 0.22	1.77 ± 0.54	2.33 ± 0.79	3.05 ± 0.89	4.55 ± 0.48	4.22 ± 1.15	4.15 ± 0.97	4.63 ± 1.32
Low dosage	1.85 ± 0.86	1.21 ± 0.75	1.01 ± 0.35	1.44 ± 0.65	2.25 ± 0.64	3.12 ± 1.11	3.88 ± 0.54	3.60 ± 0.71	3.15 ± 1.16	4.49 ± 1.23
Middle dosage	0.56 ± 0.23	0.44 ± 0.18	0.46 ± 0.51	0.62 ± 0.52	0.84 ± 0.26	1.21 ± 0.32	1.55 ± 1.02	1.84 ± 0.55	2.03 ± 0.58	2.25 ± 0.95**
High dosage	0.87 ± 0.68	0.99 ± 0.54	0.46 ± 0.29	0.11 ± 0.42	0.96 ± 0.26	0.99 ± 0.63	1.59 ± 0.19	1.85 ± 0.49	2.32 ± 0.62	2.29 ± 1.09**
Tablet	0.77 ± 0.64	0.85 ± 0.27	0.81 ± 0.57	0.91 ± 0.19	1.32 ± 0.31	1.97 ± 0.94	1.85 ± 0.44	1.75 ± 0.83	1.85 ± 0.14	2.24 ± 1.11**

_** P < 0.01 vs. model group_

**Fig. 10:**
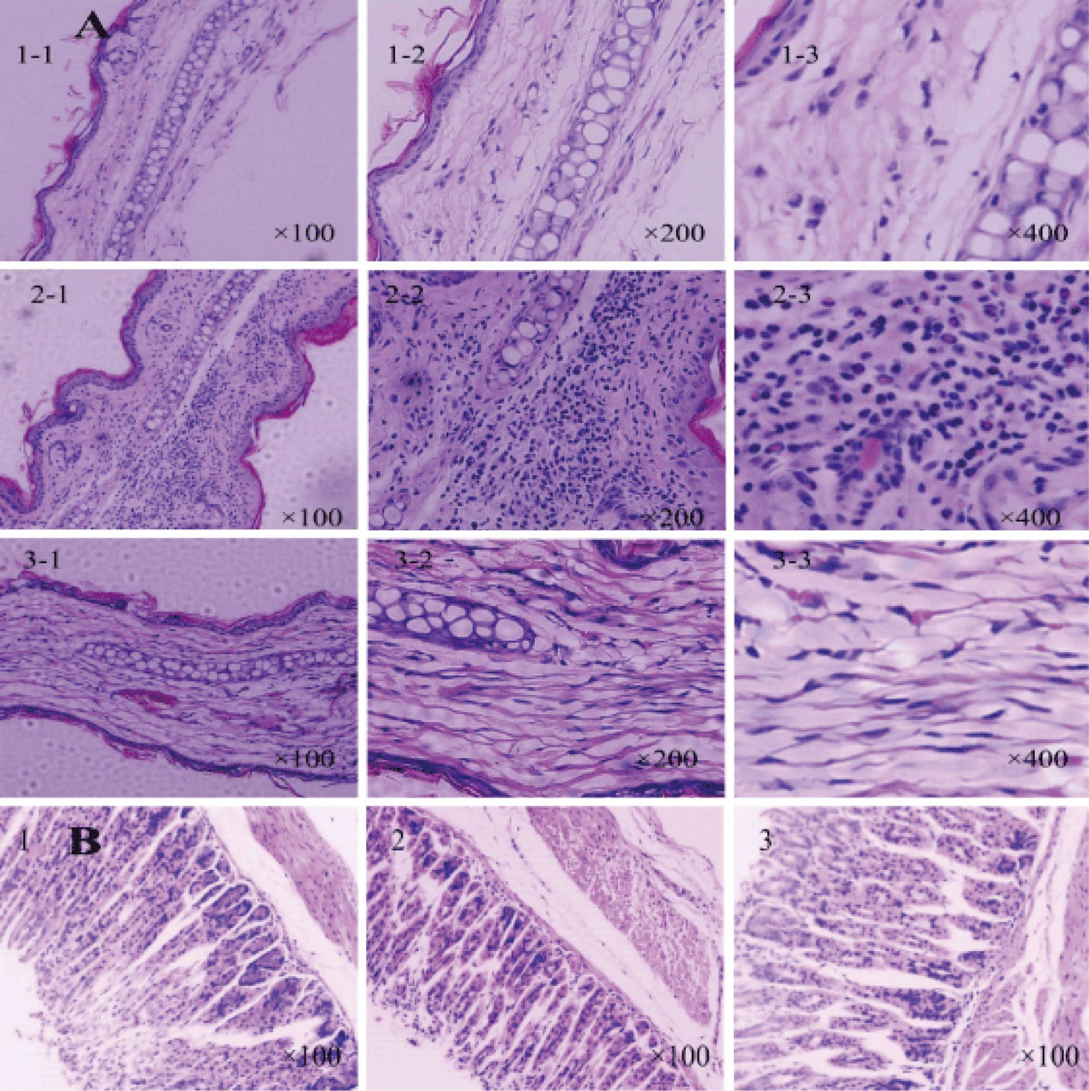
The effects of the TPL-nanoemulsion gels on the ear skin and gastric mucosa pathology of the rats (**A** ear skin pathology of the chronic dermatitis/eczema mice model, where 1–1 is the control group, 1–2 is the model group and 1–3 is the TPL-nanoemulsion gels. The magnification is 100, 200 and 400 from 1–1 to 1–3. **B** Gastric mucosa pathology of the chronic dermatitis/eczema mice model, where 1 is the control group, 2 is the model group and 3 is the TPL-nanoemulsion gels. The magnification is 100. The shooting apparatus is LEICA DFC45)

Pathological examination found that, compared with the control group, the dermis skin of the model group rats was obviously thickening (edema) and that the dermis was full of a large number of infiltrating neutrophils and lymphocytes and included visible eosinophil infiltration. The drug treatment significantly reduces edema, and the infiltration of the inflammatory cells reduces accordingly (Fig. 10) [23]. The TPL-nanoemulsion gels can reduce rat’s ear inflammation that is pathological in the eczema mice model. However, comparing the thickness of the gastric mucosa shows that *Tripterygium wilfordii* tablets have more irrigation to control than the TPL-nanoemulsion gels does, and the TPL-nanoemulsion gels causes less gastrointestinal damage than the *Tripterygium wilfordii* tablets do.

## Discussion

The sizes of TPL-nanoemulsions and nanoemulsion gels are 45.6 ± 1.7 and 62.1 ± 9.9 nm, respectively, both large than 36 nm, could be absorbed by trans-follicular routes or the aqueous pores.

The TEM image of nanoemulsions clearly reveals a core–shell structure that is an evidence of the presence of two different compositions on the same droplet. The decrease of droplet size and the relative increase of the droplet surface area may increase the function of bioactive compounds encapsulated in the nanoemulsions [24]. When droplet size is reduced, the ability of lipid interact with the biological membranes is also enhanced, thereby promoting their biological activity [25]. In this article, encapsulating TPL in nanoemulsions may improve their availability and effectiveness. The drug may be wrapped in a hydrophobic interior or hydrophilic droplet surface, or both [26]. As other literature reported [27], the smaller particle size appears to play a key role in maximizing the interface area of the encapsulated compounds to the aqueous phase. Kotyla etc. demonstrated that [28] when compared to micronized emulsion preparations of this compound nanoemulsion system improved the bioavailability of the transdermal applied delta tocopherol. Bos etc. has raised that [29] small molecule surfactants are widely used in the stability of nanoemulsion because of their absorbing ability in oil–water interface. They thought the small molecule surfactants are usually most suitable emulsifiers for producing the nanoemulsions because of their fast adsorption kinetics, low surface load and low interfacial tension. Jenning etc. also proposed the mechanism [30] that nanocarriers’ action can be attributed to their association with skin surface. The size of the small particles ensured close contact with the stratum corneum. The number of capsules penetrated into the skin can promote drug transport by changing the keratin partition coefficient of the vehicle/stratum.

Although the nanometer size and surface area plays a fundamental role in terms of increasing drug penetration [31], other nanoemulsion properties of the exciting agent type and concentration, the active pharmaceutical ingredient, as well as the nanoemulsions’ permeability membrane can also have the same effect. Other interesting nanoscale vehicles, are able to determine whether the drug’s osmotic enhancement or the effects of the drug on the skin. The surfactant on the skin surface expands the cuticle and the intact vesicle can penetrate the intact skin.

In vitro release experiments results showed drug release from nano-formulations was slightly greater than that released from the ordinary gels. This disparity is most likely due to the structure of the nano-formulations, which exhibited higher solubilizations [32]. Additionally, compared with the nanoemulsions, the nanoemulsion gels have a sudden release that is relatively slow and releases more drugs, because when the drug releases from the nanoemulsion gels, the drug comes out of the nanoemulsions before being released from the gels. During the experiment, the nanoemulsion gels released greater cumulative amounts of TPL than that released from the nanoemulsions. The enhanced drug release from the nanoemulsion gels formulations may be attributable to the drug repository that exists in this formulation that allows the nanoemulsion gels to sustain a stable release of the drug for longer and thus maintain effective drug concentrations to achieve better treatment for longer.

For the mechanisms of the in vitro percutaneous permeation experiments, our results demonstrated that transform, especially in the nanoemulsions-treated and nanoemulsion gels-treated groups, the lipids, surfactants and co-surfactants in these vehicles may erode the stratum corneum and thereby disrupt the lipid arrangement [33]. The stratum corneum of skin was loose and irregular, even resulted in a curly and deformed. The above mentioned two disruptive effects on the skin may have been exacerbated by this occlusive effect on the nanoscale lipid particles [34, 35]. Therefore, the carrier changed the skin, the interactions with the lipids and proteins in the stratum corneum, and the structure of the lipids in the skin; furthermore, the carrier improved the liquidity of the skin, changed the protein conformation and hydrated the keratin. This combination of interactions lowered the barrier function of the stratum corneum and promoted the percutaneous penetration of the contained drugs. In comparison with other vehicles, the enhanced transdermal permeation of drugs in the nanoemulsion gels and the nanoemulsions is mainly due to the more effective hydration of the stratum corneum, superior skin occlusion characteristics, and increased surface area with the skin corneocytes.

The in vivo skin-vessel synchronous microdialysis experiment was carried out to study the pharmacokinetics of TPL. The drug concentrations at the application sites of the nanoemulsions and nanoemulsion gels increased first and then decreased, which implied similar patterns of percutaneous drug absorption for both of the nanometer formulations. The nanoemulsions and nanoemulsion gels cause occlusive effects and hydration of the stratum corneum that enhanced the transdermal drug permeation. Drug absorption can be enhanced by the formation of a lipid membrane that coalesces on the stratum corneum, allowing the drug to permeate into the skin. Moreover, the drug molecules must first be released from the nanoparticles before they can be absorbed, which may cause drug levels to fluctuate in the skin.

The fluorescence analysis can be used to detected the location of a permeating molecule in skin epidermis and dermis. We found that coumarin-6 fluorescence was largely retained in the hair after laser exposure, revealing that substantial amounts of the drug passed through skin appendant organs such as hair follicles, to percutaneous absorption. At longer durations, a broad and continuous fluorescence band extended from the stratum corneum into the deeper layers of the epidermis. Fluorescence microscope showed [36] the uniform fluorescence intensity in nano-therapy, and showed the high penetrability of nanoemulsion gels through goat skin, and enhanced the residue of the drug through the skin.

## Conclusions

Nanoemulsions could be suitable for transdermal stably releasing drugs and maintaining the effective drug concentration. The TPL-nanoemulsion gels provided higher percutaneous amounts than other carriers did. The current results support the use of TPL-nanoemulsions and TPL-nanoemulsion gels as a technological platform for TPL delivery through the skin after topical administration. They influence skin, hydrating keratin and changing the structure of both the stratum corneum lipids and keratin. Both keratin hydration and the structural changes to the lipids and keratin can reduce the barrier function of the stratum corneum and thus promote the percutaneous penetration of the drug.

The fluorescence photomicrograph of skin demonstrated that the fluorescence was mainly detected in the stratum corneum at first, then with obvious evidence of diffusion into the epidermis. In addition, the fluorescence was largely retained in the hair after laser exposure, revealing that substantial amounts of the drug passed through skin appending organs to percutaneous absorption, such as hair follicles. For pharmacodynamic study, the TPL-nanoemulsion gels have a good treatment effect on dermatitis/eczema in a mice model and is expected to provide a new, low-toxicity and long-term preparation for the clinical treatment of dermatitis/eczema in transdermal drug delivery systems.

## Methods

### Materials

Triptolide was purchased from Shanghai Yuanye Biological technology co., LTD (Shanghai, China). Triptolide standards were provided by Shanghai Ronghe Pharmaceutical Technology Co., Ltd. (Shanghai, China). 1,2-propanediol, methanol and formic acid were obtained from Shanghai Ling feng Chemical Reagent Co.(Shanghai, China). Capryol90 was provided by Gattefossé Co. (Lyon, France). OP-10 was purchased from Aladdin Industrial Inc. (Shanghai, China). 2,4-dinitrochlorobenzene was purchased from Sigma-Aldrich (St. Louis, USA). 4% paraformaldehyde solution was provided by Wuhan Google biological technology Co., Ltd. (Shanghai, China). Cytokine standard, IFN-γ, IL-4 and IL-8 ELISA kits were obtained from Bender Medsystems GmbH (South San Francisco, USA). *Tripterygium wilfordii* tablets were purchased from China resources san-jiu (Yellowstone) pharmaceutical co., Ltd. (Hubei, China). All other chemicals were of analytical purity and commercially available.

### Animals

Male SD rats were of clean grade, weighed 200 ± 20 g and were approximately 2 months of age. Male ICR mice were of SPF grade, weighing 22–26 g. The animal study was approved by the Experimental Animal Center, Second Military Medical University. Animals were acclimatized for at least 7 days before the start of the study and were kept in an agreeable environment with free access to water and rodent diet.

### High performance liquid chromatography (HPLC) analysis

Triptolide samples were analyzed using an Agilent HPLC system (HP 1260, Agilent Technologies, Inc., California, USA) with a Hypersil BDS C18 column (Dalian Elite Analytical Instruments Co., Ltd, Dalian, China), (250 × 4.6 mm, 5 μm). The mobile phase consisted of a solution of methyl alcohol and water (48:52, v/v) at a flow rate of 1.0 mL/min. The column temperature was maintained at 25 °C, and the detector was set at 218 nm. Quantified samples were filtered through a 0.22 μm filter membrane (Shanghai Boguang biotechnology Co., Ltd, Shanghai, China) before automatic injection into the HPLC system. The automatic injection volume was 20 μL and the sensitivity was 0.01 AUFS.

### Liquid chromatography mass spectrometry (LC/MS) analysis

The in vivo and in vitro dialysate samples were analyzed using an Agilent 6400 Series Triple Quad LC/MS. The FAR 12.211 data analysis system was used. Chromatographic separation was achieved on an XTerraR MS C18 column (Waters, USA), (2.1 mm × 150 mm, 5 μm) maintained at 40 °C. The mobile phase consisted of methyl alcohol: 0.1% (v/v) aqueous formic acid (60:40 v/v) with a flow rate of 0.3 mL/min, and the injection volume was 3 μL. The mass spectrometer was operated in the positive mode. The spry voltage was set to 4000 kv, and the capillary temperature was set at 350 °C. The atomizer pressure was 33 psi. The selected reaction monitoring mode was multiple reactions monitoring (MRM), and quantitative analysis was performed on ions at *m/z* 361.3/105.2, minus the internal standard monitored at 361.3/147 [37]. Before automatic injection into the LC/MS, the quantified samples were centrifuged at 10,000 r/min for 5 min.

### Preparation and characterization of the nanoemulsions and nanoemulsion gels

TPL-nanoemulsions were prepared by the high-energy emulsification method with Capryol 90, OP-10 and 1,2-propanediol as the lipid, surfactant and co-surfactant, respectively. TPL was dissolved in a certain proportion of capryol 90, OP-10 and 1,2-propanediol. The required amount of the aqueous phase (distilled water) was then added to the oleic phase dropwise under seed mixing using a constant-temperature magnetic stirrer (MS-H-Pro, Rocky Hill, USA). Carbomer 940 was used as the gels matrix, and 1.5% Carbomer 940 was mixed with a suitable amount of grinding glycerin for wetting. Then, the prepared nanoemulsions were gradually added to a vessel to achieve uniform grinding. After swelling, TPL-nanoemulsion gels were acquired.

The Malvern Autosizer Nano ZS 90 inspection system (Malvern Instruments, Malvern, UK) was used to measure the average size and the zeta potential (ZP) of every prepared nanoemulsions via dynamic light scattering. The appearance of the TPL-nanoemulsions was examined by a transmission electron microscope (TEM, Libra 200, Carlzeiss, Germany). The samples were placed on a copper coated film to dry for approximately 30 min. Then, a drop of 2% phosphotungstic acid was added to the film and allowed to dry for 15 min. The samples were examined with the TEM. Then, the ultra-filtration method was used to measure the amount of free drug. Centrifugal filter tubes (10 kDa, Pall Corporation, NY, USA) were used to estimate the DL. The DL was calculated using the following equation:

1 $${\text{DL}}\% = \left( {{\text{W}}_{\text{total}} {-}{\text{W}}_{\text{free}} } \right)/{\text{W}}_{\text{lipid}} \times 100$$

where W_total_, W_free_ and W_lipid_ are the total amount of TPL in the preparation, the amount of untapped TPL and the amount of lipid used in the formulation, respectively.

### In vitro release

The dialysis method was used to assess the initial drug release. Dialysis bags (molecular weight cutoff: 10 kDa) containing 2 mL of the test formulations were firmly tied and immersed in 30 mL of PBS (pH = 7.4) containing 20% anhydrous ethanol at 37 ± 0.5 °C and stirred at 100 rpm. Aliquots of receiving liquid (1 mL) were taken from receiving pool at 0.167, 0.5, 1, 2, 3, 4, 6, 8, 12, and 24 h and then replaced with the same volume of fresh medium [38]. The withdrawn samples were centrifuged under 10,000 r/min for 5 min and measured using HPLC. All experiments were performed in triplicate.

### In vitro percutaneous permeation

Abdominal skin from the male SD rats was excised after hair was removed with depilatory, and then the subcutaneous fat and connective tissue were carefully removed. The obtained skin was rinsed with physiological saline and put in freezer for later use. In vitro permeation experiments were conducted using a Franz diffusion cell (Haimen yaohua glass instrument plant, Jiangsu, China), whose donor compartment had a diffusion area of 0.785 cm^2^, and each receptor compartment was filled with 5 mL of PBS (pH = 7.4) containing 20% anhydrous ethanol. The PBS was maintained at a temperature of 37 ± 1 °C and stirred at 300 rpm using a homemade transdermal diffusion apparatus. The saturated aqueous solution, 0.5 mL of the triptolide nanoemulsions and triptolide ordinary gels, and 0.5 g of the triptolide nanoemulsion gels were added to the donor compartment. After 1, 2, 4, 6, 8, 10, and 12 h, 1 mL of receiving liquid (and 1 mL from the receptor compartment) was removed and centrifuged at 10,000 r/min for 5 min [39]. Collected samples were analyzed using HPLC.

### Microdialysis system

The microdialysis system used was composed of a Baby Bee Syringe Drive (MD1001), a 1 mL Bee Stinger Gastight Syringe (MD0100), a Work Bee Controller (MD1000, MD-1020), a Linear Microdialysis Probe (MD-2000, LM-10) and a Vascular Microdialysis Probe (MD-2310, IV-10), all of which were purchased from BASi (West Lafayette, America).

Standard solutions were prepared by dissolving triptolide in PBS (pH = 7.4). The concentrations of the standard solutions were 100, 500, 1000 ng/mL.The in vivo and in vitro recovery rates of microdialysis probes were shown in Additional file 1.

### In vivo microdialysis studies [40]

9 SD rats were randomly divided into 3 groups, and their abdominal hairs were wiped off before the day of the experiment, according to the procedure described for the in vivo recovery validations of the implanted linear microdialysis probe and the vascular microdialysis probe. The probe was continuously perfused with PBS (pH = 7.4) at a flow rate of 3 μL/min. After an equilibration period of 60 min, the ordinary gels, the nanoemulsions or the nanoemulsion gels of triptolide was applied to the abdomen of each group of animals. Doses of 0.5 g were administered to dosing areas measuring 3 × 4 cm^2^, which was covered with polyethylene film [41]. During the experiment, the probe and the application site were kept at same level. Dialysate samples were collected in vials and were replaced every 30 min to produce 90 μL samples. Dialysis sampling was performed for 12 h. Determination of the dialysis samples was performed by LC–MS, and Kinetica 5.0 software was used to draw a pharmacokinetic curve and set the computational medicine dynamics parameters.

### Histopathological examination (HE)

Using the same procedure described for the in vitro transdermal experiments, samples of rat abdominal skin were prepared. Using a Franz diffusion cell, the skin was treated for 12 h with normal saline (as a blank control), the TPL-nanoemulsions, the blank nanoemulsions, the TPL-nanoemulsion gels, a blank nanoemulsion gels and TPL-gels. Then, the treated skin was flushed with normal saline and fixed with a 4% paraformaldehyde solution. Skin samples were embedded in paraffin, and slices were prepared and subjected to HE staining [42]. Observation with a light microscope (Leica, Buffalo Grove, Germany) was used to study the different carrier effects on the structure of the skin surface.

### Scanning electron microscopy (SEM)

Using the same procedure described for the Histopathological examination, the skin was removed and rinsed clean with distilled water. To prepare the sample, it was wrapped in moisture-absorbent paper and placed into a − 80 °C refrigerator to precool for 1 h before dehydrating for 48 h in the freeze dryer. The microstructure of the cutin layer of the skin was observed with an SEM (HITACHI S-4800, Tokyo, Japan).

### Differential scanning calorimetry analysis (DSC)

Using the same procedure described for the Histopathological examination, the skin was put into a − 80 °C refrigerator to precool for 1 h and then was dehydrated for 48 h in the freeze dryer and cut into small pieces [43]. Skin samples (4 mg) were put in an aluminium plate for DSC (Shimadzu DSC-60, Kyoto, Japan). The scanning rate was 10 °C/min and the temperature range was 30–400 °C. Analysis the DSC curve was performed to study the different carrier effects on the phase behavior of the cutin layer of the skin [44].

### Fourier Transform infrared spectroscopy (FTIR)

Using the same procedure described for the Histopathological examination, the skin was put into a − 80 °C refrigerate or to precool for 1 h and then dehydrated for 48 h in the freeze dryer and cut into small pieces. Adequately sized samples were taken to conduct the FTIR (Nicolet 6700 FT-IR, Thermo Fisher Scientific, USA) analysis. The resolution was 2 cm^−1^; the sweep number was 100; the scan range was 650–4000 cm^−1^. Analysis of the spectra revealed the different carrier effects on cuticle protein and keratin.

### Fluorescence microscopy

Coumarin-6 (Yuanye biotechnology Co., Ltd, Shanghai, China) fluorescent probes were used to mark the TPL-nanoemulsions and TPL-nanoemulsion gels. Using the same conditions described for the in vitro transdermal experiments, coumarin-6-TPL nanoemulsions and the coumarin-6-TPL nanoemulsion gels were used to treat the skin for 10, 20, 30, 40, 50 or 60 min. The skin was removed and rinsed clean with distilled water, according to the detection requirements, to study the skin. A fluorescence microscope (Leica, Buffalo Grove, Germany) was used to observe the different nanocarriers transports and dynamic distribution processes in the skin.

### Pharmacodynamic study

The mice model of dermatitis/eczema was established using a solution of DNCB acetone, a thickness gauge to measure the thickness of the ears of the ICR rat and evaluate the ear-swelling rate and the degree of inflammation. The 72 male ICR rats were randomly divided into six groups: control, model, a low dose of TPL-nanoemulsion gels (15 mg/kg), a moderate dose (45 mg/kg), a high dose (90 mg/kg) and a control group that was given *Tripterygium wilfordii* tablets (10 mg/kg). After starting the sensitization of the rats, the nanoemulsion gels groups were given different doses of TPL-nanoemulsion gels on the abdomen at 0.5 g once a day. The control and model groups were given the same amount of gels-containing medicine. The groups of *Tripterygium wilfordii* tablets were given via intragastric administration, at doses of 0.2 mL, once a day. The above groups were administered for 14 consecutive days. We measured and rated the ear thickness before and after the excitation. At 24 h after the last excitation, the ear thicknesses were measured, the mice were sacrificed, and their blood was centrifuged at 3000 r/min. The upper serum was saved in a − 70 °C refrigerator for later use. The mice ears and stomach were fixed with 4% poly formaldehyde for later use.

The ELISA method was used to measure the cytokine concentration of IFN-γ, IL-4 and IL-8 in the animal serum. The ELISA detection procedure is as follows: First, removed the microplate from the sealed bag that has been balanced to room temperature. Second, added the different concentration standards, quality control samples and experimental samples to corresponding hole (100 μL/hole). Third, sealed the reaction space with adhesive tape and incubate for 2 h at room temperature. Fourth, used the automatic washing machine to wash the microplate, added 300 μL of washing liquid to each hole, repeated 4 times and then turned the microplate upside down. Fifth, added 200 μL enzyme markers antibody to microporous, sealed the reaction hole and incubated for 2 h at room temperature. Sixth, washed the microplate again, added 200 μL chromogenic substrate, incubated in dark at room temperature for 30 min. Lastly, added 50 μL terminated liquid in each hole using a microplate reader measured the absorbance values of 450 nm within 30 min.

The mice’s ears and stomachs were treated to make paraffin sections, and then H&E staining was performed to observe the pathological changes in the tissue’s structure.

### Statistical analysis

Results are presented as the mean ± standard deviation (SD). P < 0.05 were considered to be statistically significant; P < 0.01 were considered to be extremely significant. The pharmacokinetic parameters of skin and blood were calculated using the Kinetica 5.0 software (Thermo Fisher Scientific, Inc., USA). The pharmacodynamic was calculated using Graphpad 6.02 (GraphPad Software, Inc., La Jolla, USA).

## Additional file

**Additional file 1.** Additional date of simultaneous microdialysis of the skin and vessels.

## Abbreviations

TPL: triptolide
DSC: differential scanning calorimetry analysis
FTIR: Fourier transform infrared spectroscopy
HPLC: high performance liquid chromatography
LC/MS: liquid chromatography mass spectrometry
TEM: transmission electron microscopy
RG: relative gain
RL: relative loss
HE: histopathological examination
SEM: scanning electron microscopy
PBS: phosphate buffer saline
