# Supplementary material for: Development of triptolide-nanoemulsion gels for percutaneous administration: physicochemical, transport, pharmacokinetic and pharmacodynamic characteristics
Source: J Nanobiotechnology. 2017 Dec 4;15:88. doi: 10.1186/s12951-017-0323-0 (PMC5715633; doi:10.1186/s12951-017-0323-0)
Supplement: Supplementary file 1 — Additional file 1. Additional date of simultaneous microdialysis of the skin and vessels. [file 12951_2017_323_MOESM1_ESM.doc]

**Methods**

**In vitro recovery validation**

To adopt the microdialysis probe into an in vitro recovery measuring device, we measured how the dialysis (gain) and retrodialysis (loss) perfusion rate affected the recovery of the linear microdialysis probe on the skin and the vascular microdialysis probe in vitro. The dialysis membrane portion of the linear probe or vascular probe was completely submerged in a standard solution of triptolide (100 ng/mL) in PBS (pH=7.4) at 37 ℃ in a 150mL beaker. The probe was perfused with PBS (pH=7.4) at flow rates of 3, 4 or 5 μL/min. After an equilibration period of 60 min, the dialysate was collected in a 60 µL vial and the dialysate samples were analysed using LC/MS. The relative gain (RG) was calculated as the slope of the probe regression of the drug concentration in the outgoing fluid (C _out_) as a function of the drug concentration in the ingoing fluid (C _in_):

RG = C _out_ /C _in_ × 100 (1)

For the retrodialysis studies, the probe was perfused with triptolide solution (C _in_) and the medium was replaced with PBS (pH=7.4). The drug concentration in the dialysate (C _out_) was determined, and the relative loss (RL) was calculated by the following equation:

RL = (C _in_ - C _out_) / C _in_ × 100 (2)

To adopt the microdialysis probe into an in vitro recovery measuring device, we measured how the dialysis (Gain) and retrodialysis (Loss) perfusion rate affected the recovery of the linear microdialysis probe on the skin and the vascular microdialysis probe in vitro. The dialysis membrane portion of the linear probe or vascular probe was completely submerged in standard solution of triptolide (100, 500 or 1000 ng/mL) in PBS (pH=7.4) at 37 ℃ in a 150 mL beaker. The probe was perfused with PBS (pH=7.4) at a flow rate of 3 μL/min. After an equilibration period of 60 min, the dialysate was collected in a 60 µL vial and the dialysate samples were analysed using LC/MS. RG was calculated using equation (1).

For the retrodialysis studies, the probe was perfused with a triptolide (100，500 or 1000 ng/mL, respectively) solution (C _in_) and the medium was replaced with PBS (pH=7.4). The drug concentration in the dialysate (C _out_) was determined, and RL was calculated by the equation (2).

**In vivo recovery validation in vivo correction**

Depilatory was used to wipe off the abdominal hairs of the SD rats before the day of the experiment, using an aesthesia with 25% urethane, according to body weight (0.4 mL/100 g, ip). Each rat's abdomen was fixed to the mat to keep its body temperature at approximately 37.5 ℃. A 1 cm wound was opened at the upper withers, followed by blunt separation of the jugular vein and ligation of the telocentric end of the jugular vein. Then, using the artery clamps, the proximal area of the jugular vein was nipped, and a small wound was cut in the middle of the vein. The artery clamp was loosened, and the vascular microdialysis probe (10 mm long membrane, intercept molecular weight: 5000 daltons) was inserted along the jugular vein into the atrium, followed by ligation of the probe and the jugular vein and by sewing up the wound. Then, the linear microdialysis probe (10 mm long membrane, intercept molecular weight: 5000 daltons) with an 18-g guide needle was subcutaneously implanted parallel to the vascular probe, and the guiding needle was withdrawn to leave the linear microdialysis probe membrane in the subcutaneous tissue.

After the vascular microdialysis probe was inserted into the vein and the linear microdialysis probe was implanted subcutaneously, 3 different concentrations of triptolide (C _in_=100, 500 or 1000 ng/mL) in a PBS solution (pH=7.4) were used as a perfusion fluid with the flow rate of 3 μL/min. After an equilibration period of 60 min, the dialysate was collected in a 60 µL vial and the dialysate samples were analysed using LC/MS. The RL was calculated by equation (2).

Equation (3) was used to correct the in vivo recovery. R is the recovery for thedialysis method and D is the recovery for the retrodialysis method.

$\left(\frac{R}{D}\right)in\text{ }vivo=\left(\frac{R}{D}\right)in\text{ }vitro$ (3)

**Results and discussion**

**In vivo simultaneous microdialysis of the skin and vessels**

Before researching the in vivo effects, we first used the method of dialysis method (gain) and retrodialysis method (loss) perfusion rate to examine the recovery of the linear microdialysis probe of the skin and the vascular microdialysis probe of the vessels in vitro. The experimental results(Tab 1) show that with an increase in the perfusion rate, the probe recovery gradually reduces; when the perfusion rate is 3 μL/min, the linear microdialysis probe on the skin and vascular microdialysis probe of the vessels both have high recovery rates, and the measured recovery rates of the dialysis and retrodialysis methods are basically identical. Thus, a perfusion rate of 3 μL/min is preferred.

**Tab 1 Effect of flow on the *in vitro* recovery determined by the gain and loss methodswiththe linear and vascular microdialysis probes（n=5）**

| Flow（μL/min） | Liner recovery (%) | | Vascular recovery (%) | |
| --- | --- | --- | --- | --- |
|  | Gain method | Loss method | Gain method | Loss method |
| 3 | 36.46±0.65 | 35.32±3.48 | 31.95±2.12 | 29.38±2.96 |
| 4 | 30.70±1.33 | 31.28±1.38 | 23.18±1.44 | 20.77±2.55 |
| 5 | 26.68±1.43 | 28.69±3.73 | 22.11±1.75 | 18.81±1.25 |

The experimental results (Tab 2) show that, in different drug concentrations, both the linear microdialysis probe on the skin and the vascular microdialysis probe measured essentially the same probe recoveries, indicating that drug concentration had no significant effect on the probe recovery.

**Tab 2** Effect of triptolide concentration on the in vitrorecovery determined by the gain and loss methodswith the linear and vascular microdialysis probes (n=5)

| Concentration | Liner recovery (%) | | Vascular recovery (%) | |
| --- | --- | --- | --- | --- |
| (ng/mL) | Gain method | Loss method | Gain method | Loss method |
| 100 | 36.46±0.65 | 35.32±3.48 | 31.95±2.12 | 29.38±2.96 |
| 500 | 39.67±0.37 | 40.74±4.66 | 31.83±1.00 | 30.14±1.29 |
| 1000 | 33.97±1.14 | 35.33±1.76 | 36.62±0.21 | 32.50±1.24 |

Linear and vascular microdialysis probes of the in vivo recoveries (Table 3) show that with a perfusion rate of 3 μL/min, the recovery at the different concentrations is basically consistent. The loss method of the linear microdialysis probe has an in vivo recovery of 21.18% ± 3.7%, and the loss method of the vascular microdialysis probe has an in vivo recovery of 23.39% ± 2.48%. Equation (3) was used to revise the in vivo recovery.

**Tab 3** Effect of triptolide concentrations on the in vivo recovery determined by the loss method from the linear and vascular microdialysis probe (*n*=5)

| Concentration（*n*g/mL） | 100 | 500 | 1000 |
| --- | --- | --- | --- |
| Linear recovery（%）  Vascular recovery（%） | 19.16±3.8  23.40±3.12 | 24.59±3.25  26.59±2.54 | 19.79±3.95  20.19±1.77 |

By the result of in vitro recovery research, the measured recovery rate of dialysis method and retrodialysis method for triptolide basically consistent, so correction of the in vivo recovery can be used in calculation drug concentration in the process of pharmacokinetic. Calculating equation:

C = C _dialysis_/R _in vivo_  (4)

**Responses to comments**

We have done the additional experiments at the referee’s suggestion. We have done the additional experiments at the referee’s suggestion. The shaved mice abdominal skins were treated for 12 h with the normal saline, the TPL-nanoemulsions, the blank nanoemulsions, the TPL-nanoemulsion gels, a blank nanoemulsion gels and TPL-gels. After 12 hours, cleaning the mice abdominal with distilled water carefully and the mice were free to eat. After 2 days, the mice were sacrificed. We divided the experiment site of the mice abdominal skins into two parts. One of them was flushed with normal saline and fixed with a 4% paraformaldehyde solution. Skin samples were embedded in paraffin, and slices were prepared and subjected to HE staining. The other part of skins were removed and rinsed clean with distilled water. To prepare the sample, it was wrapped in moisture-absorbent paper and placed into a -80 ℃ refrigerator to precool for 1 h before dehydrating in the freeze dryer for 48 h. The microstructures of the cutin layer of the skins were observed with an SEM. The photographs of Histopathological microscopic slides and microstructure of the mice skins were in Additional file Figure 1.


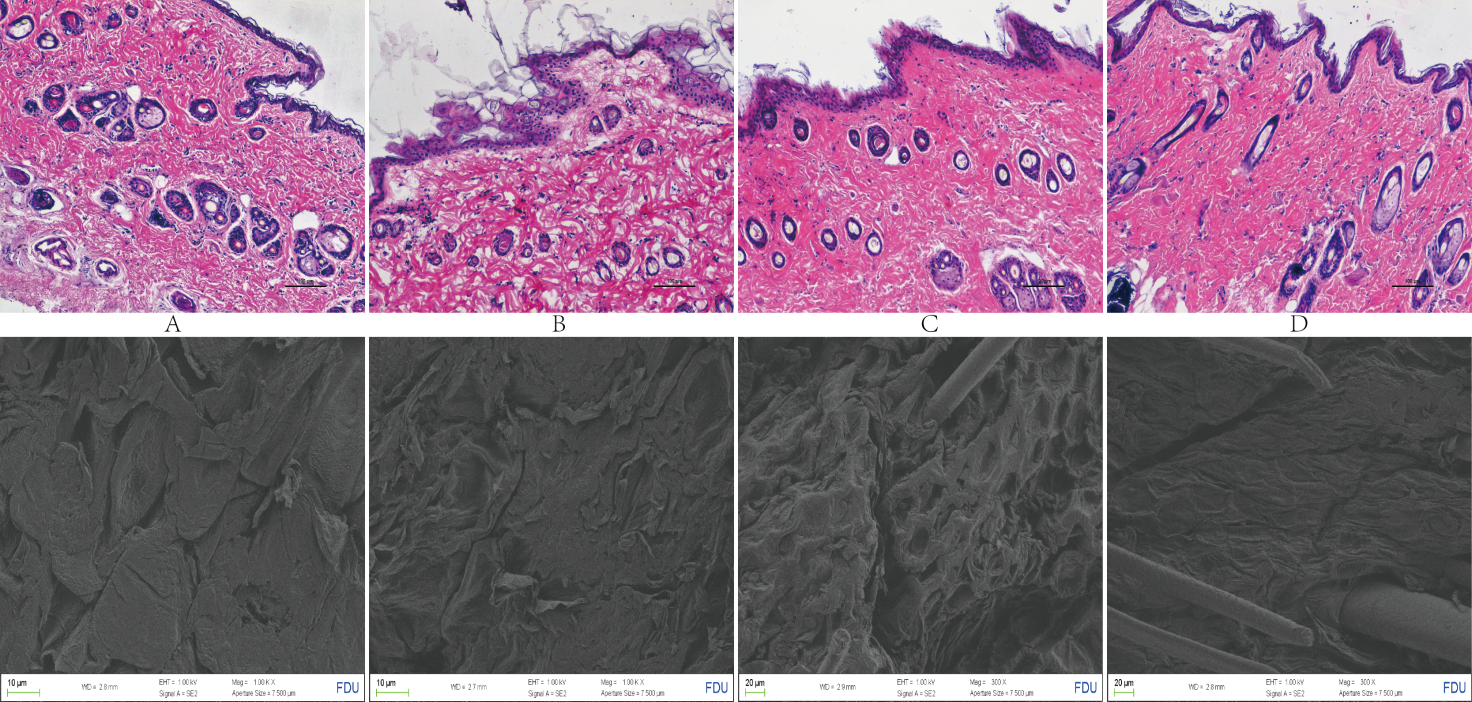


**Figure 1** Skin samples with TPL-nanoemulsions-treated (A), TPL-nanoemulsion gels-treated (B), TPL-nanoemulsions-treated 2 days after withdrawal (C), TPL-nanoemulsion gels-treated 2 days after withdrawal (D) obtained via HE dyeing method and SEM (×1000 magnification).

As shown in photographs, the stratum corneum of mice abdominal skinswere healing after drug withdrawal for 2 days, its means this effect on the skin stratum corneum is transient. And the transient damage of skin stratum corneum promoted the percutaneous penetration of the contained drugs, which confirmed our original findings.
